# Displacement of Racially and Ethnically Minoritized Groups after the Installation of Stormwater Control Measures (i.e., Green Infrastructure): A Case Study of Washington, DC

**DOI:** 10.3390/ijerph181910054

**Published:** 2021-09-24

**Authors:** Alisha Yee Chan, Ji-Young Son, Michelle Lee Bell

**Affiliations:** 1 Department of Chemical and Environmental Engineering, School of Engineering and Applied Science, Yale University, New Haven, CT 06511, USA; 2 School of the Environment, Yale University, New Haven, CT 06511, USA; jiyoung.son@yale.edu (J.-Y.S.); michelle.bell@yale.edu (M.L.B.)

**Keywords:** displacement, environmental justice, stormwater control measures, green infrastructure, race, ethnicity, gentrification

## Abstract

Stormwater control measures (SCMs) (i.e., green infrastructure) are advantageous methods of stormwater management. However, studies suggest that urban greening may be associated with gentrification, displacing racially/ethnically minoritized groups due to increased housing costs and loss of feelings of belonging. We studied displacement of racially/ethnically minoritized groups after SCM installation in Washington, DC. We compared the change in percentage of persons in racial/ethnic groups at the Census block group level with varying levels of SCM installation (i.e., area-weighted SCM count at 300 m buffer). We stratified findings by SCM type, pre-installation income, and SCM size. DC installed a higher density of SCMs in areas with a higher percentage of Black and/or Hispanic/Latino residents. Nonetheless, findings suggest SCM installation is associated with displacement of Black residents. The percentage of residents who are Black decreased by 2.2% [95% Confidence Interval: 1.7, 2.7] and 4.1% [95% Confidence Interval: 3.4, 4.8] after low and high levels of SCM installation, respectively. In turn, the change in percentage of residents who are White increased with increasing levels of SCM installation. Compared to ecological studies on SCMs, studies about social impacts are scarce. This research intends to help optimize SCM installations so more residents can enjoy their health, economic, and ecological benefits.

## 1. Introduction

Impervious surfaces in urban areas often result in high peak flow rates of stormwater runoff and lead to problems such as stream bank erosion and flash floods [1,2,3]. Stormwater control measures (SCMs) are methods of stormwater management that capture, retain, and/or treat stormwater runoff using ecologically sustainable methods such as infiltration, detention, and/or bioretention [4]. SCMs were previously referred to as “Best Management Practices” [5] and include “Green Infrastructure” and “Green Stormwater Infrastructure”, which are structural forms of SCMs. SCMs function by increasing storage, promoting groundwater recharge, lowering peak flow rates, decreasing the volume, and improving the water quality of stormwater runoff [6,7]. In addition to advantages for stormwater management, SCMs may have benefits such as enhanced urban aesthetics and creation of jobs through construction and maintenance [8,9,10]. SCMs that include vegetation may have similar benefits to urban green space such as decreased mortality, improved mental health, improved air quality, and reduced urban heat island effect [8,9,11,12,13,14,15,16].

Racially/ethnically minoritized groups, defined by the journal, Lancet, in 2020 as groups that have a higher likelihood of pre-existing conditions or live in poverty due to an existing racial and/or ethnic social hierarchy [17], often face disproportionate public health burdens from environmental exposures. Within urban communities, these groups generally reside in areas with the highest levels of air pollutants and the urban heat island effect [18,19,20,21]. Additionally, racially/ethnically minoritized groups may be more vulnerable to the health hazards related to stormwater events for several reasons. These persons often reside in higher-risk areas prone to flooding and other hazards [22]. Additionally, racially/ethnically minoritized persons living in poverty may live in less structurally sound housing that is more susceptible to flood damage and mold [23]. Racially/ethnically minoritized groups overall have less access to health care than other persons, which could affect their ability to respond in the case of illness or injury related to stormwater [24]. However, flood protection measures often face cost-benefit analyses that favor populations with high income and a high percentage of White and non-Hispanic/Latino residents [25].

SCM implementation strategies in relation to Black and Hispanic/Latino residents may be paradoxical. Previous research has revealed that though greening methods can provide ecological, health, and social benefits, they can also increase housing costs and property values [26,27,28]. Scholars commonly refer to this phenomenon as “green gentrification”, the influx of wealthier new residents and outflux of low-income residents, often of racially/ethnically minoritized groups, where greening initiatives are implemented [29]. A previous study revealed that present-day greenspace accessibility is less in neighborhoods with lower Homeowners’ Loan Corporation grades [30]. This study suggests that redlining, the discriminatory practice from the 1930s of denying services such as loans and mortgages to certain racial/ethnic groups, may continue to impact urban greening accessibility for racially/ethnically minoritized groups in the present [30]. Previous studies on green gentrification found that racially/ethnically minoritized groups that lived in the location pre-installation often feel a lower sense of community and belonging after the introduction of urban greening due to the social changes that followed [31,32,33,34]. The historical practice of redlining, the increased housing costs, and the feelings of loss of belonging may all be drivers for the displacement of racially/ethnically minoritized groups after urban greening.

It is unclear whether the displacement of residents of racially/ethnically minoritized groups seen in previous studies after the implementation of urban greening also occurs after the installation of SCMs. Taguchi et al.’s review on the possible consequences of green stormwater infrastructure highlighted the lack of studies on the potential gentrification associated with the installation of specific types of green stormwater infrastructure (e.g., infiltration, filtering systems) [35]. In that review, the authors discuss the importance of considering environmental justice during green stormwater infrastructure implementation as it can lead to increases in property values, displacement of pre-existing communities, and feelings of loss of community belonging or identity [26,35].

The goal of our study was to assess the relationships between SCM installation and the displacement of racially/ethnically minoritized groups. First, we identified the socio-demographic areas in which a higher density of SCMs (e.g., count per subsewershed area) have been installed. Then, we studied whether White, Black, and/or Hispanic/Latino displacement is associated with SCM installation density and stratified this analysis by SCM type, pre-installation median annual household income, and SCM size. Such research is needed on this environmental justice issue to ensure that these groups can reap the stormwater, health, economic, and social benefits of SCMs rather than be displaced by them.

## 2. Methods and Materials

### 2.1. Study Site

Our study site, Washington, DC, has a dense population and high percentage of impervious area (38.5%) [36,37]. DC has a high percentage of Black residents (46.0%) compared to that of the entire United States (13.4%). The percentage of Hispanic/Latino residents in DC is 11.3%, lower than that of United States at 18.5% [37]. DC was chosen as an appropriate study site because of its high level of urbanization, population density, and abundance and diversity of SCMs [38].

### 2.2. Data

The locations of SCMs were obtained from the DC Department of Energy and the Environment. The dataset included SCM characteristics such as type, installation date, coordinate location, and size (ft^2^). SCM types found in DC are listed and described in the supplementary materials (Table S1) [38,39,40,41,42,43,44,45,46,47,48,49,50,51]. There were too few de/retention, grass channels, stormwater planters, stream restoration, swales, wetlands, and open channels installed during the study period for appropriate statistical analysis specific to those SCM types. The “other” category, which included proprietary practice, patented/manufactured forms of SCMs, was also not examined separately due to sample size and heterogeneity among included types. Changes in demographics for Census block groups with a specific type of SCM installed were compared to Census block groups without installation of that type of SCM. However, de/retention, grass channels, stormwater planters, stream restoration, swales, and wetlands were included in our analysis in aggregate to study “Vegetated SCMs”; open channels were included when studying “Non-vegetated SCMs”, and all SCMs were included when studying “Total SCMs”.

Socio-demographic data including percentage of residents that are White, Black, and Hispanic/Latino as well as median annual household income and percentage of housing that is rented were acquired from the American Community Survey (ACS) 5-year data at the Census block group level for DC. Socio-demographic data were acquired for eight 5-year intervals within the time period of 2007 to 2018. We organized these datasets into four time periods of two 5-year datasets each, centered around the years 2011 to 2014. The four time periods are listed below, where T0 represents the first 5 years of each period (T0) and T1 represents the last 5 years of each period (T1).

Period 1 (P1): T0: 2007–2011 and T1: 2011–2015 (Center Year: 2011)

Period 2 (P2): T0: 2008–2012 and T1: 2012–2016 (Center Year: 2012)

Period 3 (P3): T0: 2009–2013 and T1: 2013–2017 (Center Year: 2013)

Period 4 (P4): T0: 2010–2014 and T1: 2014–2018 (Center Year: 2014)

Changes in socio-demographics across time were calculated as the difference between the socio-demographics in the first 5-year interval, represented by T0, and those in the second 5-year interval, represented by T1 for each period. SCMs were matched to a period based on their year of installation to assess socio-demographics before and after installation (e.g., SCMs installed in 2011 were matched to P1). At the center year of these four periods (i.e., 2011 in P1, 2012 in P2, 2013 in P3, and 2014 in P4), SCMs of various types and densities were installed in some, but not all, Census block groups. Throughout this paper, we explore the differences in the mean of the aggregated change in socio-demographics in Census block groups from time intervals T0 to T1 for all four studied periods as various densities and types of SCMs are installed at the center year each period. In other words, we compare whether socio-demographics changed in the 5-year period after SCM installation compared to the previous 5 years, considering different characteristics of SCMs such as density and type. We compared these temporal changes in socio-demographics to that of Census block groups within SCM installations for the same timeframes. Only new SCMs (non-cumulative) installations during the center years (2011–2014) were considered in our analysis. SCMs installed prior to 2011 and after 2014 were not included in our study.

### 2.3. Assessment of SCM Intensity and Statistical Analysis

As Census block group boundaries can change over time, we used area weighting to convert all 5-year ACS Census block group dataset boundaries to match the Census block group boundaries of the 2014–2018 dataset. Among the 450 Census block groups, three Census block groups in the 2014–2018 dataset had undefined median annual household incomes and therefore were removed from the analysis.

First, we studied where SCMs were installed in relation to pre-installation socio-demographic variables (i.e., characteristics of the community for the 5-year period before SCMs were installed, T0) by performing a linear regression between SCM density, calculated as the number of SCMs per area, of each SCM type and socio-demographic. Next, we studied the associations between change in racial/ethnic percentage and SCM installations (i.e., comparison of community socio-demographics before and after installation) using a difference-in-difference method. We defined SCM installation exposure density based on the area-weighted number of SCMs using a 300 m buffer. The metric considers the possibility of more than one SCM and accounts for “exposure” from multiple SCMs. We generated a 300 m buffer, an approximate 5-min walk [52], around each SCM location. Many buffer-zones overlapped, signifying that some locations are exposed to multiple SCMs. We then calculated the area- and overlapping-weighted percentage of each Census block group that was within the SCM buffer zones. This procedure was modeled after earlier work [53]. We then compared the change in racial/ethnic percentages of Census block groups by level of SCM installation exposure density levels (SCM-IEDL): 0 (no SCMs installed), low (first tertile: SCM-IEDL ≤ 0.995), medium (second tertile: 0.995 < SCM-IEDL ≤ 2.09), and high (third tertile: SCM-IEDL > 2.09).

Data on SCMs of each type were overlaid on the Census datasets to reveal the SCMs of each type that were installed within each Census block group during each installation year. The change in racial/ethnic percentages between T0 and T1 in Census block groups that had at least one SCM installation of a given type in the center year and that in Census block groups that had no SCM installations of that given type in the center year were both calculated. The changes in racial and ethnic percentages between T0 and T1 in Census block groups that had at least one SCM installation of the given type within each period were then aggregated among all periods and averaged as was the changes in racial/ethnic percentages between T0 and T1 in Census block groups that had no SCM installations of that given type within each period. Separate analyses were performed for each SCM type.

We examined whether changes in race/ethnicity after SCM installation differed by socio-economic status using a variable for pre-installation (T0) median annual household income. We also investigated whether the temporal changes in racial/ethnic group percentages differed for Census block groups with installations of SCMs in comparison to Census block groups without such installations to disentangle the influence of SCMs from other temporal trends in socio-demographics. For this analysis, we stratified by pre-existing (T0) median annual household income levels for low (first tertile), medium (second tertile), and high (third tertile). Different tertiles were calculated for each of the four periods. For Census block groups with SCM installations, the mean aggregated change in demographics was calculated as the difference between the characteristics of T0 and that of T1. The “pre-existing” (or pre-installation) income was that of T0. For Census block groups without SCM installations, we compared the median annual household income in T0 to that of T1. Analysis was performed separately for each of the four periods, P1 to P4, and then data of all four periods were combined. We performed this analysis for SCMs overall (after aggregating SCMs of all types) and a secondary analysis for SCMs by individual type.

Data on the size (i.e., area) of the SCMs were incomplete for most types of SCMs. However, the dataset provided the surface area size (ft^2^) of 97.1% of green roofs in DC. A buffer radius of 300 m applied to the location of the green roofs was overlaid to the ACS datasets. If over half of a green roof’s buffer area was outside of the study area of DC, it was removed from the analysis. The aggregated change in racial/ethnic percentage within the 300 m buffer radius of small (first tertile: ≤1085 ft^2^), medium (second tertile: >1085 ft^2^, ≤4048 ft^2^), and large (third tertile: >4048 ft^2^) green roofs in the 5-year interval before installation (T0) and that in the 5-year interval after installation (T1) were compared.

ArcGIS 10.8.1 (Esri, Redlands, CA, USA) was used to assess the data [54]. R Studio 1.2.1335 (RStudio, Boston, MA, USA) was used for statistical analysis [55,56].

## 3. Results

### 3.1. Descriptive Statistics

**Figure 1:**
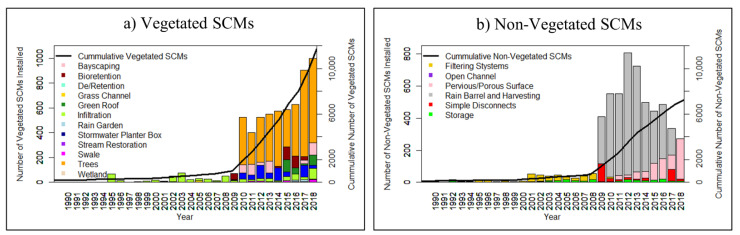
Installation of (**a**) Vegetated and (**b**) Non-vegetated stormwater control measures (SCMs) from 1990 to 2018 in Washington, DC.

In recent years, DC has installed more vegetated SCMs than non-vegetated SCMs (Figure 1). The most common type of vegetated SCM that was installed in DC, since 2010, is trees (Figure 1a), while the most common type of non-vegetated SCM that has been installed, since 2009, is rain barrels/rainwater harvesting (Figure 1b). The installation of rain barrels/rainwater harvesting has decreased in recent years and the installation of pervious/porous pavement has become more popular (Figure 1b). Washington, DC installed 3546 vegetated and 3017 non-vegetated SCMs between 2011 and 2014. Correlations among socio-demographic variables in DC can be found in the Supplementary Materials (Tables S2 and S3).

### 3.2. SCM Installation and Pre-Installation Socio-Demographics

There was a lower density of SCMs placed in areas with a higher percentage of residents who are White and a higher density of SCMs installed in areas with higher percentage of residents who are Black and/or Hispanic/Latino (Table 1). Among most SCM types, except storage, a higher density of SCMs were placed in Census block groups with higher pre-installation socio-economic status as indicated by a higher median household incomes and lower percent of housing that is rented (Table 1).

### 3.3. Displacement and SCM Installation Exposure Density

**Figure 2:**
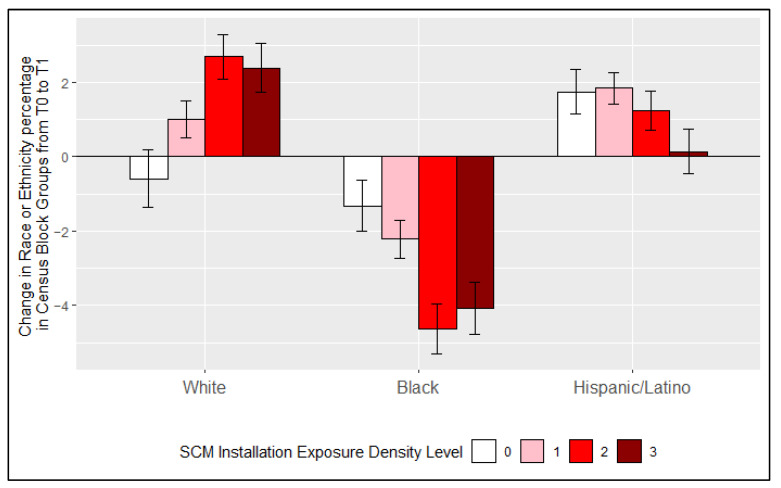
Change in percent of residents who are White, Black, and Hispanic/Latino in Census block groups from the 5-year interval before stormwater control measure (SCM) installation (T0) to the 5-year interval after SCM installation (T1) with varying levels of SCM installation exposure density. Error bars represent the 95% confidence intervals.

Figure 2 shows the change in the percent of the population that is White, Black, or Hispanic/Latino comparing the pre-and post-installation periods (T0 and T1, respectively) by three levels of SCM density (low, medium, and high) and for Census block groups without SCM installations, for which T0 and T1 correspond to the time periods of the installations in other block groups. For Census block groups with SCM-IEDL of 0 (i.e., no SCMs were installed) the percentage of residents who are White declined, with a −0.59% [95% CI: −1.4%, 0.17%] change from T0 to T1. However, for the Census block groups with SCM installations (i.e., SCM-IEDL of low to high), the percentage of residents who are White increased after installation. The percentage of residents who are White increased more for Census block groups with more SCMs installed, with higher increases for medium (+2.7% [95% CI: 2.1%, 3.3%]) or high SCM-IEDL (+2.4 [95% CI: 1.7%, 3.1%]) compared to that of low SCM-IEDL (+1.0% [95% CI: 0.52%, 1.5%]). The changes in percent of residents who are White in Census block groups were not statistically different between those with medium SCM-IEDL and those with High SCM-IEDL (Figure 2). The percentage of residents who are Black decreased over time (between T0 and T1) for Census block groups regardless of the SCM-IEDL but decreased more for Census block groups with SCMs installed. This decrease was higher in magnitude for medium (−4.6% [95% CI: −5.3%, −4.0%]) and high SCM-IEDL (−4.1% [95% CI: −4.8%, −3.4%]) compared to low (−2.2% [95% CI: −2.7%, −1.7%]) and 0 SCM-IEDL (−1.3% [95% CI: −2.0%, −0.63%]). The changes in percentage of residents who are Black in Census block groups were not statistically different between SCM-IEDL of 0 and low SCM-IEDL or between medium SCM-IEDL and high SCM-IEDL (Figure 2). The percentage of residents who are Hispanic/Latino in Census block groups increased from T0 to T1, regardless of the SCM-IEDL. The changes in percent of residents who are Hispanic/Latino in Census block groups were not statistically different between varying SCM-IEDL levels (Figure 2).

A sensitivity analysis was included in the supplementary files revealing the change in the population count, rather than change in percentages, from T0 to T1 of residents who are White, Black, and/or Hispanic/Latino in areas where SCMs were and were not installed (Figure S1). The population count of Black, Hispanic, and White increased regardless of whether SCMs were installed. However, the population count that is Black increased less and population that is White increased more if SCMs were installed.

### 3.4. Displacement and SCM Type

**Figure 3:**
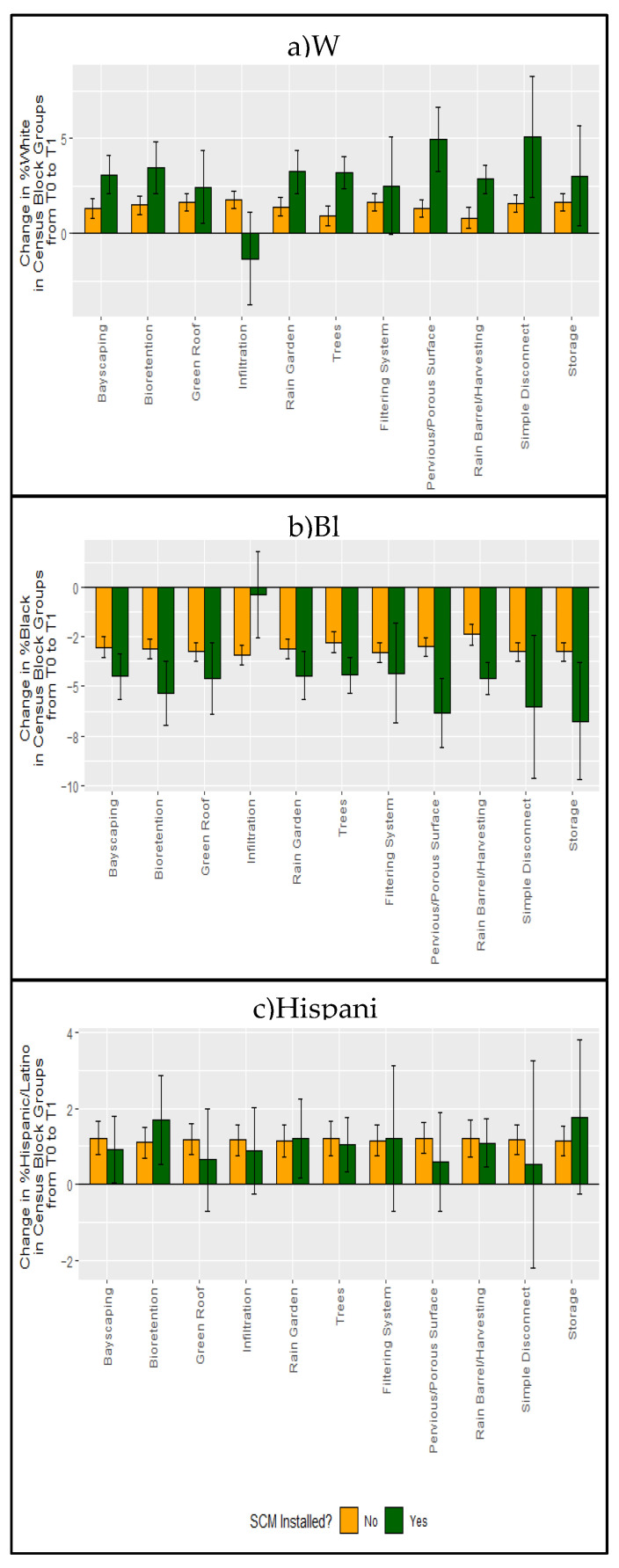
Change in percent of residents who are (**a**) White, (**b**) Black, and (**c**) Hispanic/Latino in Census block groups from the first 5-year interval (T0) to the last 5-year interval (T1) with or without stormwater control measure (SCM) installation of specific types. Error bars represent 95% confidence intervals.

We did not observe noticeable differences between the change in percentage of the population that is White, Black, or Hispanic/Latino after the installation of vegetated compared to non-vegetated SCMs (Figure S2). However, we did find differences by specific type of SCM. For all SCM types, the percentage of the population that is White increased and the percentage that is Black decreased more for Census block groups with SCM installations of a specific type compared to Census block groups without SCM installation of that type, except for infiltration (Figure 3).

The percentage of residents who are White significantly increased more, and the percentage of residents who are Black residents significantly decreased more from T0 to T1 if bayscaping, bioretention, rain gardens, trees, pervious/porous pavement, rain barrels/rainwater harvesting, and simple downspout disconnections were installed compared to that in Census block groups without installation of those SCM types. The percentage of residents who are Black also decreased more from T0 to T1 if installations of green roofs and storage were implemented between time intervals T0 and T1 compared to Census block groups for which no SCMs of that type were implemented (Figure 3a,b). The percentage of residents who are White decreased from T0 to T1 in Census block groups that implemented infiltration whereas this value increased in Census block groups with no infiltration installed. Additionally, there was a smaller decrease in the percentage of residents who are Black from T0 to T1 in Census block groups that had implemented infiltration than that in Census block groups that did not implement infiltration between T0 and T1. The differences in the change in percentage of residents who are Hispanic/Latino in Census block groups from T0 to T1 in Census block groups that had implemented SCMs of a specific type was not statistically significant from those for Census block groups that had not implemented that type of SCM, for all SCM types considered (Figure 3c).

### 3.5. Displacement and Income

**Figure 4:**
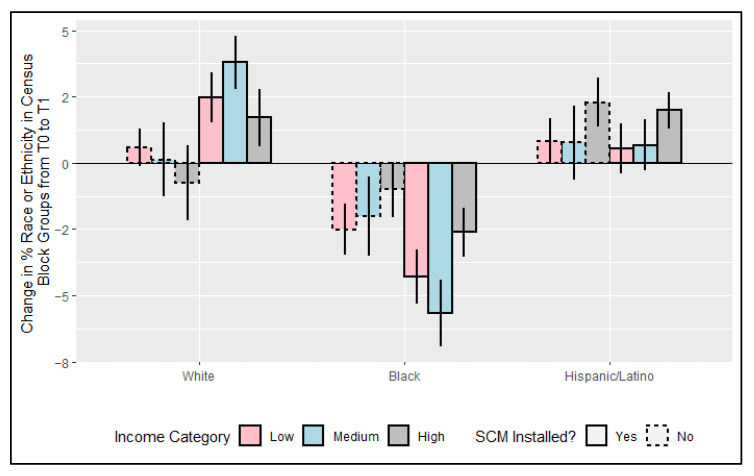
Change in percent of residents who are White, Black, and Hispanic/Latino in Census block groups with and without stormwater control measure (SCM) installations with pre-existing income categories in the first (low), second (medium), and third (high) tertiles. Error bars represent 95% confidence intervals. SCMs were installed in some, but not all Census block groups between the year T0, the first 5-year interval, and T1, the last 5-year interval.

We examined whether the change in race/ethnicity associated with SCM installation differed by the communities’ income. We considered pre-existing (T0) annual median household income for Census block groups. The percentage of residents who are White in Census block groups with low- and medium-income categories increased more for Census block groups with SCMs installed. The change in the percentage of residents who are White in Census block groups without SCMs installed was +0.58% [95% CI: −0.12%, 1.3%], +0.12% [95% CI: −1.3%, 1.5%, and −0.76 [95% CI: −2.2%, 0.64%] for Census block groups with low, medium, and high income, respectively (Figure 4).

When SCMs were installed, the change in percentage of residents who are White for Census block groups with low-, medium-, and high-income was 2.5% [95% CI: 1.5%, 3.4%], 3.8% [95% CI: 2.8%, 4.8%], and 1.7% [95% CI: 0.62%, 2.8%], respectively (Figure 4). The percentage of residents who are Black in Census block groups of all three income categories decreased when no SCMs were installed (−2.5 [95% CI: −3.5%, −1.5%], −2.0% [95% CI: −3.5%, −0.53%], and −1.0% [95% CI: −2.0%, 0.038%] for low, medium, and high income, respectively), but the decrease was larger in magnitude if SCMs were installed (−4.3% [95% CI: −5.3%, −3.3%], −5.7% [95% CI: −6.9%, −4.4%], and −2.6% [95% CI: −3.5%, −1.7%] for low, medium, and high income, respectively) (Figure 4). For Census block groups with SCMs installed, the largest increase in percentage of residents who are White and largest decrease in percentage of residents who are Black was for Census block groups in the medium-income category (Figure 4). There were no statistically significant differences between the change in percentage of residents who are Hispanic/Latino in Census block groups that installed SCMs and that in Census block groups that did not install SCMs among the three income categories (Figure 4). The change in the racial and ethnic percentage with SCM installations of individual types can be found in the supplementary materials (Figure S3). The difference in rented housing and in median year housing that was built between T0 and that in T1 for Census block groups with and without SCM installation for all periods, stratified for each income category, is shown in the supplementary materials (Table S4).

### 3.6. Displacement and SCM Size

**Figure 5:**
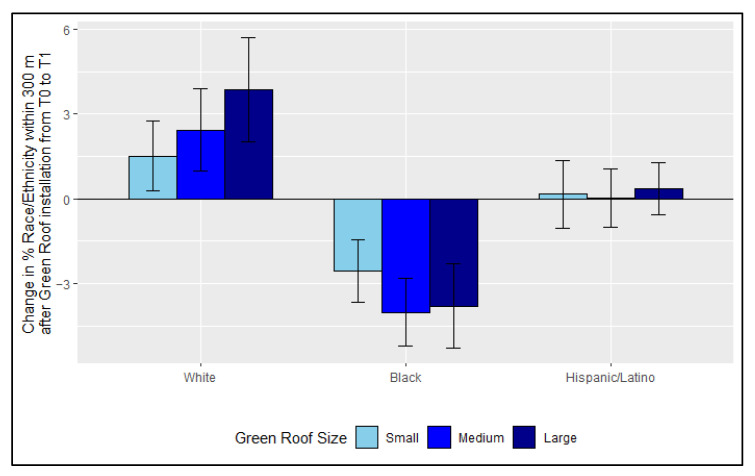
Change in percent of residents who are White, Black, and Hispanic/Latino surrounding green roof installation of varying sizes. Error bars represent 95% confidence intervals. SCMs were installed in some, but not all Census block groups between the year T0, the first 5-year interval, and T1, the last 5-year interval.

We investigated whether changes in race/ethnicity from T0 to T1 in relation to installation of green roofs differed by size of green roof. The decrease in the percentage of residents who are Black and increase in the percentage of residents who are White was generally larger for Census block groups with installations of medium or large size green roofs compared to small green roofs, although comparisons were not significantly different (Figure 5).

## 4. Discussion

Our findings suggest that for Washington, DC, SCMs are more often placed in areas with a higher percentage of residents who are Black and/or Hispanic/Latino and lower percentage of residents who are White. These areas may have increasing structural development, during which SCMs may be installed to meet stormwater regulations. Our results show that SCM installation is associated with decreases in the percentage of the population that is Black and increases in the percentage that is White, which suggests the displacement of racially minoritized residents in DC. Areas with higher SCM installation densities have higher magnitudes of displacement of Black residents. Therefore, though SCMs are placed in areas with a higher percentage of residents within racially and ethnically minoritized groups, some of these groups may not fully benefit from the SCMs because they are instead displaced. This same trend in change in racial demographics was observed after stratifying by SCM type, except for the SCM type of infiltration. We conjecture that infiltration may not have been associated with the displacement of Black residents because though it is a vegetated SCM, it has a lower profile compared to the other vegetated SCMs as it often does not contain plants other than grass [39]. The installation of SCMs was associated with a decrease in the percentage of the population that is Black, suggesting the displacement of Black residents, regardless of the Census block group’s pre-existing income level. While this study cannot quantify the number of persons who moved in, moved out, or stayed for each Census block group, and therefore cannot prove displacement, the study findings do demonstrate changes in racial/ethnic composition of the communities. The results indicate that such changes are not the sole result of scenarios in which racial/ethnic minority subpopulations were not displaced in combination with many more White subpopulations moving into the area. Rather, these findings suggest that both the number of White subpopulations increased and the number of racial/ethnic minorities decreased with SCM installation compared to communities without SCM installation.

Results suggest that smaller green roofs may be less associated with the displacement of Black residents compared to larger green roofs, although this result was not statistically significant. A previous study found no associations between greening project size and level of gentrification [57]. However, the trend seen in our results was similarly observed in multiple studies about gentrification surrounding large-scale and small-scale urban greening projects [26,58]. Benefits of smaller green roofs may include less maintenance, less stringent regulations, and less management [26,58] Although this result is not statistically significant, it suggests that the size of SCMs may have varying impacts on the level of displacement of racially minoritized groups. However, as there exists opposing arguments about the impact of size of urban greening and displacement, additional research is needed on this topic. In addition, as small SCMs often mitigate less stormwater than larger ones, which is often the original purpose of implementing an SCM, additional research is needed to study whether multiple small sized SCMs may have a different impact on displacement than fewer large sized SCMs would.

The installation of many types of SCMs in Washington, DC may have increased in popularity due to incentives from the RiverSmart Homes Program, which piloted in 2008. RiverSmart Homes is a District-wide program that offers installation incentives to homeowners for the implementation of SCMs such as rain barrels, rain gardens, bayscaping, pervious pavement, and trees to reduce the effects of stormwater runoff [59]. The program recognizes Washington, DC’s high percentage of impervious surfaces and aims to reduce the impacts of stormwater runoff by treating and infiltrating stormwater on site to recharge groundwater, diminish stormwater pollutants to streams, and prevent erosion. The RiverSmart Homes Program encourages the implementation of vegetative SCMs such was bayscaping and rain gardens to provide additional benefits to air quality, reduction of the urban heat island effect, and habitats for native species [59]. Additionally, the RiverSmart Homes Program may have made SCMs more affordable to homeowners. For instance, shade trees, currently the most popular form of SCMs installed in DC (Figure 1), can be provided for free in DC’s residential properties through the RiverSmart Homes Program.

As previously mentioned, studies have suggested that racially/ethnically minoritized groups may be displaced after urban greening, not just because of the raise in property prices, but also because of the culture and social changes and loss of sense of community that may accompany greening [31,32,33,34]. The processes underlying such demographic shifts are complex and relate to historical and ongoing cultural, societal, and economic factors including redlining and structural racism, with links to human health and urban infrastructure [60,61]. As such, previous studies have revealed that involving the community through participation and education may stimulate a greater sense of community belonging [62,63,64]. Community cohesion and unity was found to be predicted by the degree of community participation, rather than the length of residency or demographic characteristics [64]. Green stewardship can strengthen resiliency at individual, interpersonal, and community levels because it builds confidence, strengthens social ties, broadens social networks, and provides the community with trusted residential leaders [62,63]. A study that interviewed residents of Detroit, Michigan had expressed support for green infrastructure projects. However, many interviewees had felt an unclear responsibility towards maintaining the structures due to inadequate communication between the residents and the city government and a lack of proper governance [65]. Interview participants from another study from Atlanta, Georgia had revealed a similar lack of trust and negative perceptions from the community about the local government. As such, greening projects created by government agencies had lower degrees of participation and were less well received [66]. These studies suggest the importance of collaboration and involvement of the community when implementing greening projects. As such, more research should be conducted to study whether community involvement can help to reduce displacement of racially/ethnically minoritized groups after urban greening.

Our analysis was limited by the available data. SCMs that were implemented at the beginning of a year were treated the same as SCMs that were implemented at the end of the year. We used 5-year time intervals to represent the timeframe before and after installation. The relationship between SCMs and demographics over time is still unknown, such as the impacts of one year post installation versus five or ten years. We used 5-year time intervals due to the lack of yearly socio-demographic data at the Census block group level. An analysis of the lagged effect of SCM installation on displacement by year may help us determine the temporal trends of SCM installation’s impact on the displacement of residents of racially/ethnically minoritized groups. Another limitation we faced was that some SCM types (de/retention, grass channels, stormwater planters, stream restoration, swales, wetlands, and open channels) were not abundant enough for appropriate statistical analyses by those types individually. Additionally, factors such as natural disasters or shifts in the US economy may also impact both SCM installation and demographic trends. Results from Washington, DC may not be generalizable to other areas. This city is the center of many government agencies, but the district is also unique in that it has a high percentage of residents who are Black, high population density, and high levels of impervious surfaces. Significant results in this study should not be treated as causal, but rather as associations.

## 5. Conclusions

The findings support the hypothesis that SCM installation is associated with the displacement of residents from racially/ethnically minoritized groups. Even though SCMs were more often installed in areas with a higher percentage of residents who are Black and/or Hispanic/Latino, these persons, especially Black residents, may not fully benefit from their ecological, health, social, and economical advantages because they are displaced. Additional research is needed to study whether involving residents in the implementation of SCM and installing smaller SCMs may lessen the displacement impact on racially/ethnically minoritized persons. To the best of our knowledge, this is the first study to assess the impact of SCM installation on the displacement of racially and ethnically minoritized groups. Compared to ecological studies on SCMs, studies about the societal impacts of SCMs are scarce. Through this research, we do not intend to discourage the future installation of SCMs as they have many benefits, but instead we aim to help further optimize the installation of SCMs to ensure that their benefits can be reaped by more residents.

## Figures and Tables

**Table 1:** Change in SCM installation density (SCM count per km^2^) in 2014 per interquartile range (IQR) increase of pre-installation socio-demographics variables.

	Bayscaping	Bioretention	Green Roof	Infiltration	Rain Garden	Trees	Total Vegetated	Filtering System	Pervious/Porous Surface	Rain Barrel/Harvesting	Simple Disconnect	Storage	Total Non-Vegetated	Total SCMs
White (%)	−0.63 *	−0.41 *	0.26	−0.04	−0.25 *	−0.80	−1.82 *	0.04	−0.12	−2.08 *	−0.11	−0.02	−2.29 *	−4.10 *
Black (%)	0.53 *	0.35 *	−0.30	0.05	0.21	0.41	1.20	−0.06	0.02	1.75 *	0.10	0.01	1.83 *	3.00 *
Hispanic/Latino (%)	0.26 *	−0.03	0.05	−0.03	0.03	1.13 *	1.40 *	0.06 *	0.20 *	0.70 *	0.00	0.02	0.98 *	2.43 *
Annual Median Household Income ($1000)	0.04	−0.13	−0.07	−0.01	0.18 *	0.82 *	0.82 *	−0.02	−0.04	0.59 *	0.06	−0.06 *	0.55	1.22
Rented Housing (%)	−0.97 *	0.08	0.20	0.02	−0.75 *	−3.44 *	−4.86 *	0.07	−0.11	−4.49 *	−0.24 *	0.09 *	−4.69 *	−9.26 *
N (# of SCMs)	583	358	174	91	383	1924	3546	78	223	2570	94	52	3017	6789

Note: * *p* < 0.05. Vegetated SCMs include bayscaping, bioretention, green roof, infiltration, rain garden, and trees. Non-vegetated SCMs include filtering system, previous/porous surface, rain barrel/harvesting, simple disconnect, and storage.

## Data Availability

The demographic data presented in the study are publicly available from the United States Census Bureau American Community Survey’s 5-year Estimates as TIGER/Line Shapefiles and can be found here: [https://www.census.gov/geographies/mapping-files/time-series/geo/tiger-data.html] (accessed on 15 December 2020). The locations of stormwater control measures, also known as best management practices, that support the findings of this study are available from the Department of Energy and the Environment at Open Data DC, https://opendata.dc.gov/datasets/best-management-practices (accessed on 9 October 2020).

## Supplementary Materials

The following are available online at https://www.mdpi.com/article/10.3390/ijerph181910054/s1, Table S1: List and Descriptions of Vegetated and Non-vegetated SCM types in Washington, DC, Table S2: Pearson’s correlation coefficients among socio-demographic characteristics in Washington, DC Census Block groups (5-year period: 2014–2018), Table S3: Pearson’s correlation coefficients among change in socio-demographic characteristics in Census block groups from the 5-year period: 2010–2014 to the 5-year period: 2014–2018, Table S4: Change in rented housing and median year housing was built from T0 to T1 in Census block groups with pre-existing median household income categories in the first, second, and third tertiles, Figure S1: Change in population count of residents from T0 to T1 who are White, Black, and/or Hispanic/Latino in Census block groups with and without SCM installation., Figure S2: Change in percent of residents that are (a) White, (b) Black, and (c) Hispanic/Latino in Census block groups with varying levels of non-vegetated and vegetated SCM installation exposure density, Figure S3: Change in percent of residents who are (a) White, (b) Black, and (c) Hispanic/Latino in Census block groups with and without SCM installations of each type with pre-existing income categories in the first, second, and third tertiles.
