# Supplementary material for: Displacement of Racially and Ethnically Minoritized Groups after the Installation of Stormwater Control Measures (i.e., Green Infrastructure): A Case Study of Washington, DC
Source: Int J Environ Res Public Health. 2021 Sep 24;18(19):10054. doi: 10.3390/ijerph181910054 (PMC8508036; doi:10.3390/ijerph181910054)
Supplement: Supplementary file 1 [file ijerph-18-10054-s001.zip › ijerph-1367958-supplementary.pdf]

Supplementary Information

Displacement of Racially and Ethnically Minoritized Groups after the Installation of Stormwater Control Measures (i.e., Green Infrastructure): A Case Study of Washington, DC

Alisha Yee Chan[1,*], Ji-Young Son[2], Michelle L. Bell[2]

[1]Yale University, Chemical and Environmental Engineering, New Haven, CT, USA

[2]Yale University, School of the Environment, New Haven, CT, USA

*Address correspondence to Alisha Yee Chan, Yale University, Chemical and Environmental Engineering. 17 Hillhouse Ave 5th floor, New Haven, CT 06511. Email: alisha.chan@yale.edu.

List of Tables and Figures

Page Number	Description
**S1**	Table S1: List and Descriptions of Vegetated and Non-vegetated SCM types in Washington, DC.
**S3**	Table S2: Pearson's correlation coefficients among socio-demographic characteristics in Washington, DC Census Block groups (5-year period: 2014-2018).
**S4**	Table S3: Pearson's correlation coefficients among change in socio-demographic characteristics in Census block groups from the 5-year period: 2010-2014 to the 5-year period: 2014-2018.
**S5**	Figure S1: Change in population count of residents from T0 to T1 who are White, Black, and/or Hispanic/Latino in Census block groups with and without SCM installation.
**S6**	Figure S2: Change in percent of residents that are a) White, b) Black, and c) Hispanic/Latino in Census block groups with varying levels of non-vegetated and vegetated SCM installation exposure density.
**S7**	Figure S3: Change in percent of residents who are a) White, b) Black, and c) Hispanic/Latino in Census block groups with and without SCM installations of each type with pre-existing income categories in the first (Low), second (Medium), and third (High) tertiles.
**S8**	Table S4: Change in rented housing and median year housing was built from T0 to T1 in Census block groups with pre-existing median household income categories in the first, second, and third tertiles.

Table S1: List and descriptions of vegetated and non-vegetated SCM types in Washington, DC. (N) symbolizes SCMs that were not individually studied in the main text. [1,2]

	SCM Type	Description
Vegetated	**Bayscaping**	Landscaping practice that replaces grass with plants native to the Chesapeake Bay. These plants have deep rooting systems that absorb more stormwater. Currently only in the Washington, DC area.[3]
	**Bioretention**	Collection of stormwater through layers of sand, gravel soil, mulch, vegetation, and ponding to decrease volume and contaminants of runoff.[4]
	**Constructed Wetland**	Artificial wetland containing soil, vegetation, and microorganisms that are commonly used to treat stormwater, wastewater, and/or greywater.[5]
	**De/Retention Basin (N)**	A pond or structure used as an artificial lake to hold stormwater. Retention basins maintain a pool of water and often include vegetation while detention basins are often temporary flood control structures.[2]
	**Grass Channel (N)**	Grassed open channel that are designed to capture, treat and/or convey stormwater runoff.[1]
	**Green Roof/Ecoroof**	Layers of soil, vegetation and ponding used to perform bioremediation and decrease peak flowrate of stormwater on rooftops.[6] Types of green roofs include extensive green roofs, which are often low profile and inaccessible to the public and intensive green roofs, which often have a wider variety of plants and are accessible to the public.
	**Infiltration**	Grassed ditches that collect stormwater and allow it to infiltrate the ground.[1]
	**Rain Garden**	Garden commonly consisting of native plants designed to catch stormwater runoff from impervious surfaces and promote groundwater recharge.[7]
	**Shade Trees**	Large trees with widespread, dense canopies. The large leaves hold stormwater.[8]
	**Stormwater Planters (N)**	Small, contained vegetated area that uses bioretention techniques to treat stormwater. Planter boxes often allow high volumes of ponding and are often located at the end of building's downspout.[1]
	**Stream Restoration (N)**	The use of rocks, logs, and native plants to slow down stormwater flowrate, restore the natural flow pattern of a stream, and reduce erosion to protect nearby habitats and restore aquatic ecosystems.[9]
	**Swale (N)**	Area of land that has a lower elevation than surrounding area. Vegetated at varying intensities.[10]
Non-Vegetated	**Filtering System**	Capture and temporarily store stormwater on site as it passes through a filter bed of sand media. Often used for highly impervious sites such as parking lots.[1]
	**Pervious/Porous Surfaces**	Pavement that allows stormwater to infiltrate into the ground below[11,12]
	**Rain Barrell**	Placed at the bottom of stormwater downspouts to catch and store stormwater for later use.[13]
	**Simple Disconnect**	Disconnection of downspouts from pipes or impervious area to allow stormwater to infiltrate instead.[14]
	**Storage**	Includes rooftop storage and underground detention (large underground vaults designed to temporarily hold stormwater runoff). Storage practices do not treat stormwater quality and can only act as a form of volume management for large storm events.[1]
	**Proprietary Practice (N)**	Patented/manufactured stormwater treatment practices.[1]

The descriptions of various types of SCMs are listed in Table S1. This list includes SCMs, symbolized by (N) that were installed in DC but were not individually studied in the main text. SCMs labeled as proprietary practice were not studied individually because of the wide range of characteristics of SCMs that were labeled proprietary practice. The other types of SCMs with the

symbol (N) were not individually studied due to limited abundance of installations from 2011-2014. However, these SCM types were re-included when studying “Vegetated SCMs”, “Non-vegetated SCMs,” and/or “Total SCMs.” The SCMs listed in Table S1 are limited to SCMs that were installed from 2011-2014 in DC and may not include SCMs that were installed during other years nor in other cities. Also note that some SCM types may intersect. For example, a green roof may use bioretention techniques to treat stormwater. We grouped and labeled the SCMs based on how the Department of Energy and Environment labeled the data when it was provided to us.

Table S2: Pearson's correlation coefficients among socio-demographic characteristics in Washington, DC Census Block groups (5-year period: 2014-2018). **$^*p<0.05$**

	Percent White	Percent Black	Percent Hispanic/Latino	Median Annual Household Income
Percent Black	**-0.96***			
Percent Hispanic/Latino	**0.12***	**-0.32***		
Median Annual Household Income	**0.71***	**-0.67***	0.06	
Percent Rented Housing	**-0.23***	**0.18***	0.08	**-0.61***

The correlations between socio-demographic characteristics in Census block groups in DC reveal that there is a strong negative correlation between the percentage of residents who are White and the percentage of residents who are Black. Additionally, the median annual household income is higher in Census block groups with a higher percentage of White residents and a lower percentage of Black residents. Rented housing is less common in Census block groups with higher median household incomes (Table S2).

Table S3: Pearson's correlation coefficients among change in socio-demographic characteristics in Census block groups from the 5-year period: 2010-2014 to the 5-year period: 2014-2018 (includes all Census block groups, regardless of whether SCMs were installed). *p<0.05

	Δ Percent White	Δ Percent Black	Δ Percent Hispanic/Latino	Δ Median Annual Household Income
Δ Percent Black	**-0.67***			
Δ Percent Hispanic/Latino	0.09	**-0.44***		
Δ Median Annual Household Income	**0.18***	**-0.17***	0.01	
Δ Percent Rented Housing	-0.01	0.00	0.05	**-0.19***

Table S3 reveals that as the percentage of residents who are White increases between the 5-year period: 2010-2014 and the 5-year period: 2014-2018, the percentage of residents who are Black decreases and the medium annual household income increases. As the percentage of residents who are Black increase between the two periods, medium household income decreases. (Table S3).

Figure S1 reviews the change in population counts of the studied races and ethnicity from T0 to T1 for Census block groups with and without SCM installation.

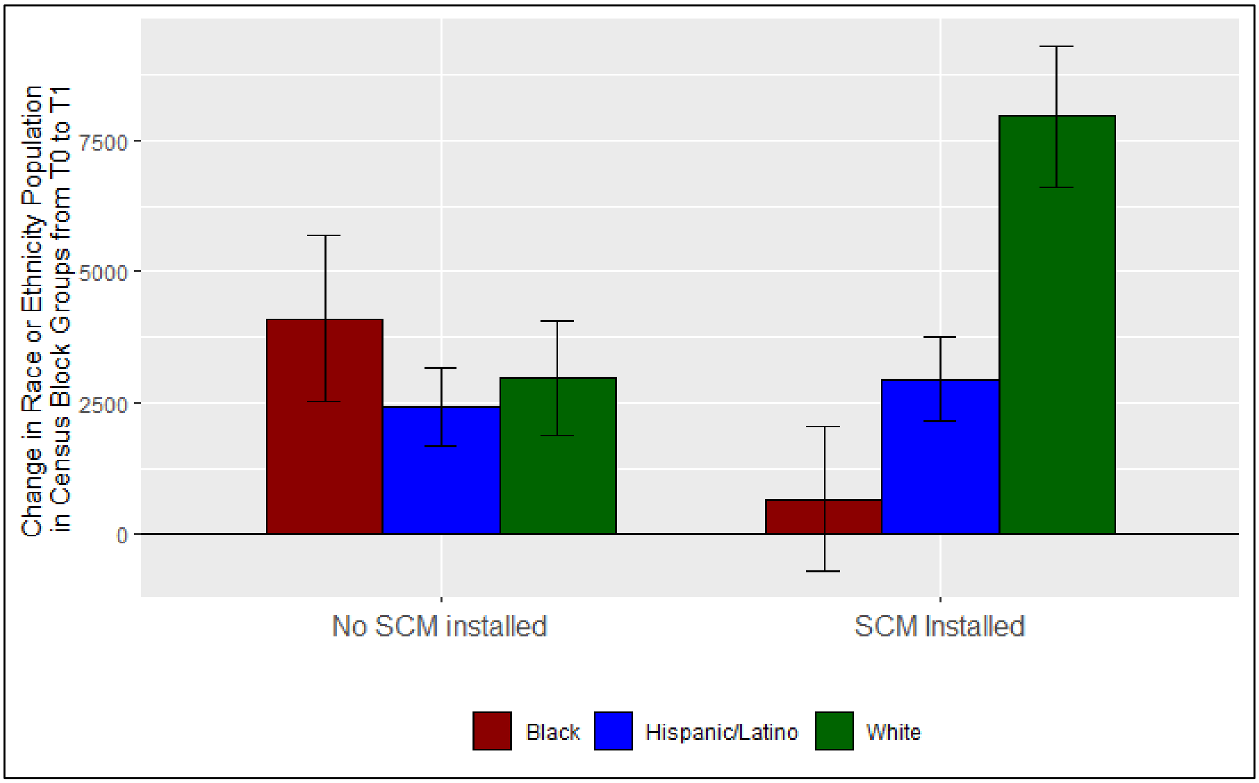

Figure S1: Change in population count of residents from T0 to T1 who are White, Black, and/or Hispanic/Latino in Census block groups with and without SCM installation. SCMs were installed in some, but not all Census block groups between the year T0, the first 5-year interval, and T1, the last 5-year interval.

Figure S2 reveals that the change in the percentage of reisdents who are White, Black, and/or Hispanic/Latino after the installation of non-vegetated SCMs is not siginficantly different from that after the installation of vegetated SCMs.

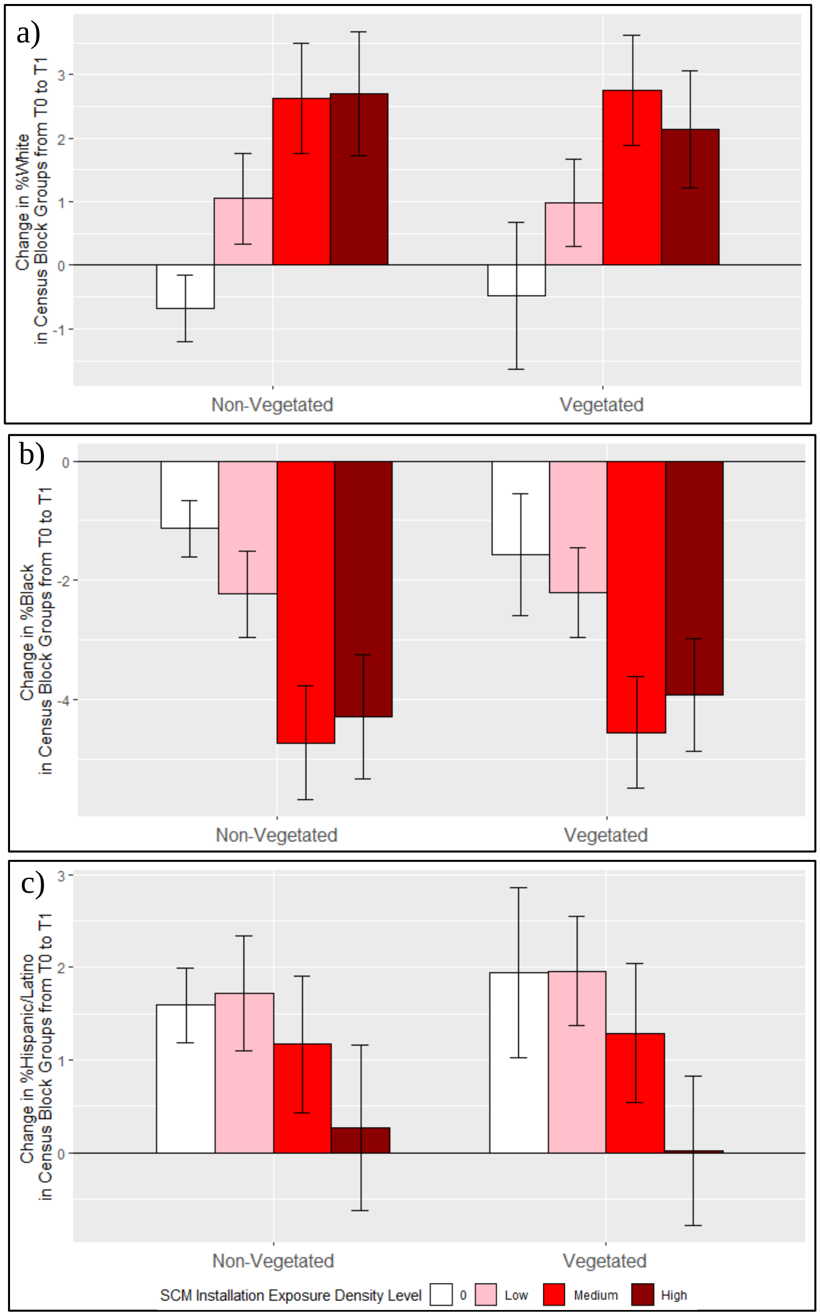

Figure S2: Change in percent of residents who are a) White, b) Black, and c) Hispanic/Latino in Census block groups from T0 to T1 with varying levels of non-vegetated and vegetated SCM installation exposure density. Error bars represent 95% confidence intervals. SCMs were installed in some, but not all Census block groups between the year T0, the first 5-year interval, and T1, the last 5-year interval.

Figure S3 suggests that, regardless of income category, SCM installations of each type were associated with an increase in the the change in percentage of residents who are White and decrease in the percentage of residents who are Black from T0 to T1 except for infiltration. Census block groups within the highest tertile of median household income that installed infiltration was associated with an increase in the percentage of residents who are Black whereas the lack of installation of infiltration was asoociated with a decrease. The installation of infiltration in areas with high median household incomes may be associated with less displacement of Black residents.

Note that the SCM type: Simple Disconnect was not included in Figure S3 because it was not installed in enough abudnace among each income category (<1% Census block groups for the category).

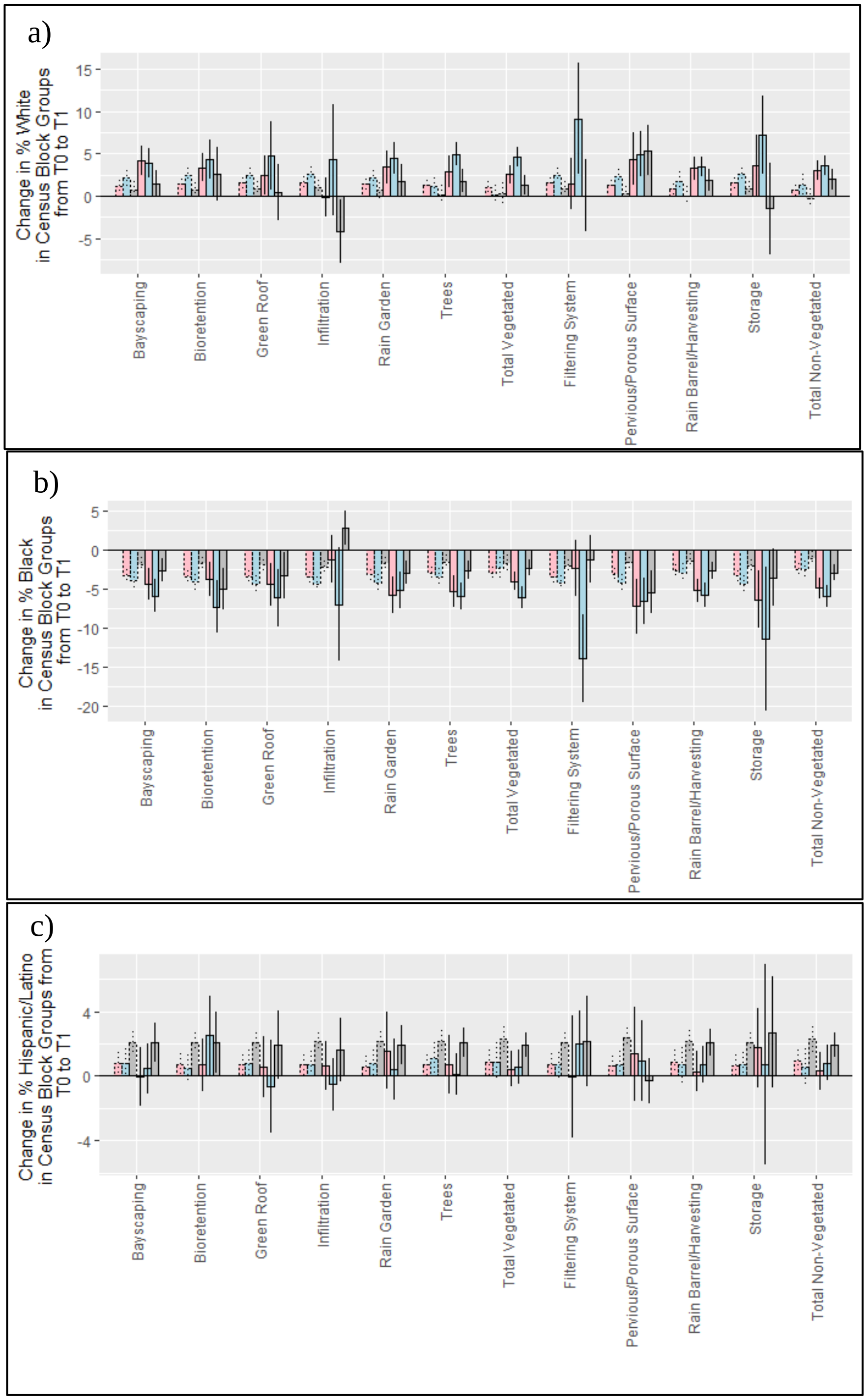

Figure S3: Change in percent of residents who are a) White, b) Black, and c) Hispanic/Latino in Census block groups with and without SCM installations of each type with pre-existing income categories in the first (“1” or Low), second (“2” or Medium), and third (“3” or High) tertiles. Error bars represent 95% confidence intervals. SCMs were installed in some, but not all Census block groups between the year T0, the first 5-year interval, and T1, the last 5-year interval.

Table S4: Change in rented housing and median year housing was built from T0 to T1 in Census block groups with pre-existing median household income categories in the first (Low), second (Medium), and third (High) tertiles.

SCM Installed?	Income Category	Δ Rented Housing [SE]	Δ Median Year Housing Built [SE]
Yes	Low	0.21 [0.59]	2.2 [0.66]
	Medium	-0.27 [0.54]	1.57 [0.40]
	High	1.68 [0.51]	1.42 [0.45]
No	Low	0.71 [0.54]	2.09 [0.55]
	Medium	-0.29 [0.84]	0.52 [0.62]
	High	2.22 [0.71]	1.14 [0.52]

Table S4 reveals that a larger amount of development occurred in areas with medium-income category that installed SCMs than in areas with medium-income category that did not install SCMs. Table S4 also reveals that the percentage of rented housing has been decreasing in Census block groups with a medium-income catgory while the percentage of rented housing has been increasing in areas with low- and high-income cagetories. We speculate that the installation of SCMs in Census block groups medium income may have a greater impact on the displacement of Black residents because there is a shift towards owned housing rather than rented housing so residents, rather than landlords, attend to the installation and maintanence of SCMs.

References

(1) District Department of the Environment; Center for Watershed Protection. *Stormwater Management Guidebook*; District Department of the Environment, Watershed Protection Division: District of Columbia, 2020.

(2) United States Environmental Protection Agency. Green Infrastructure https://www.epa.gov/region8/green-infrastructure (accessed 2021 -03 -31).

(3) DDOE. *Stormwater Management Guidebook*; District Department of the Environment, Watershed Protection Division: District of Columbia, 2013.

(4) United States Environmental Protection Agency, Office of Water. Storm Water Technology Fact Sheet: Bioretention. 1999.

(5) United States Environmental Protection Agency, O. Constructed Wetlands https://www.epa.gov/wetlands/constructed-wetlands (accessed 2021 -03 -31).

(6) United States Environmental Protection Agency. Soak Up the Rain: Green Roofs https://www.epa.gov/soakuptherain/soak-rain-green-roofs (accessed 2020 -10 -20).

(7) US EPA. Soak Up the Rain: Rain Gardens https://www.epa.gov/soakuptherain/soak-rain-rain-gardens (accessed 2020 -02 -25).

(8) District Department of Energy and the Environment. RiverSmart Homes - Shade Tree Planting https://doee.dc.gov/service/riversmart-homes-shade-tree-planting (accessed 2021 -03 -31).

(9) Yochum, S. *Guidance for Stream Restoration*; TN-102.4; United States Department of Agriculture, Forest Service, National Stream and Aquatic Ecology Center: Fort Collins, CO, 2018.

(10) United States Environmental Protection Agency, Office of Water. Storm Water Technology Fact Sheet: Vegetated Swales. 1999.

(11) United States Environmental Protection Agency. Soak Up the Rain: Permeable Pavement https://www.epa.gov/soakuptherain/soak-rain-permeable-pavement (accessed 2020 -02 -25).

(12) United States Environmental Protection Agency, Office of Water. Storm Water Technology Fact Sheet: Porous Pavement. 1999.

(13) District Department of Energy and the Environment. RiverSmart Homes - Rain Barrels https://doee.dc.gov/service/riversmart-homes-rain-barrels (accessed 2021 -03 -31).

(14) United States Environmental Protection Agency. Soak Up the Rain: Disconnect / Redirect Downspouts https://www.epa.gov/soakuptherain/soak-rain-disconnect-redirect-downspouts (accessed 2020 -02 -25).
